# Prospects and Limitations Related to the Use of MicroRNA as a Biomarker of Epilepsy in Children: A Systematic Review

**DOI:** 10.3390/life11010026

**Published:** 2021-01-04

**Authors:** Beata Rzepka-Migut, Justyna Paprocka

**Affiliations:** 1 Department of Pediatric Neurology and Pediatrics, St. Queen Jadwiga’s Regional Clinical Hospital No. 2, 35-301 Rzeszów, Poland; beata-rzepka@o2.pl; 2 Department of Pediatric Neurology, Faculty of Medical Science in Katowice, Medical University of Silesia, 40-752 Katowice, Poland

**Keywords:** microRNA, miRNA, epilepsy, children, biomarker, prognosis, diagnosis

## Abstract

Epilepsy is one of the most common neurological diseases in children. There is an unmet need for new objective methods that would facilitate and accelerate the diagnostic process, thus improving the prognosis. In many studies, the participation of microRNA in epileptogenesis has been confirmed. Therefore, it seems to be a promising candidate for this role. Scientists show the possibility of using microRNAs as diagnostic and predictive biomarkers as well as novel therapeutic targets. Children with epilepsy would benefit particularly from the use of this innovative method. However, the number of studies related to this age group is very limited. This review is based on 10 studies in children and summarizes the information collected from studies on animal models and the adult population. A total of 136 manuscripts were included in the analysis. The aim of the review was to facilitate the design of studies in children and to draw attention to the challenges and traps related to the analysis of the results. Our review suggests a high potential for the use of microRNAs and the need for further research.

## 1. Introduction

Epilepsy is a chronic neurological disease with a heterogeneous clinical picture, characterized by the occurrence of asynchronous spontaneous and recurrent neural discharges. According to the World Health Organization (WHO), the disease affects more than 50 million people worldwide and the incidence in pediatric patients is estimated at 33–82/100,000 per year [1]. The diagnosis of epilepsy is based on the clinical picture presented by the patient that can be difficult to verify when there are no witnesses to the seizure. The diagnosis is also made on the basis of test results (electroencephalogram—EEG; neuroimaging studies). The experience of a physician is also of crucial importance in this respect [2]. Since the whole diagnostic process is largely subjective, objective studies are sought to prevent misdiagnosis. Scientists have high hopes for the discovery of the genetic background of epilepsy, which could be a reliable diagnostic tool. However, despite knowing nearly 200 genes associated with the disease in most patients, the genetic background is still unknown [3]. Rapid and accurate diagnosis, combined with effective treatment, is particularly important in the group of pediatric patients, as it would help to prevent severe sequelae of the disease such as exposure to mental disorders, including anxiety disorders, depression [4], delayed psychomotor development or cognitive disorders. Despite great effort, approximately 30% of patients are resistant to antiepileptic drugs, prompting further studies on the molecular basis of epilepsy and the implementation of targeted treatment.

One of the variants considered by scientists in the pathomechanism of epilepsy is the participation of microRNAs (miRNAs), i.e., short non-coding RNAs, approximately 18 to 25 nucleotides in length, acting at the post-transcriptional stage by being coupled to complementary mRNA, which results in degradation of the transcript or inhibition of protein synthesis. The expression of many genes is modulated by miRNAs. Single miRNAs target multiple proteins. At the same time, proteins can be regulated by a large number of miRNAs. More than 30% of the genes encoding proteins are regulated with miRNA [5]. Thus, manipulation of a single miRNA can interfere with a single pathway or simultaneously with many pathways [6]. The brain is the organ with the highest number of miRNAs, probably due to a complex mechanism of action [7]. Many studies have proven the contribution of miRNA to cell proliferation and migration, neuronal apoptosis and neuroinflammation.

Extracellular vesicles (EVs) are small membrane vesicles released by different cells into the microenvironment in response to physiological and pathological processes. In terms of diameter, there are three main classes: ectosomes (micro-vesicles), exosomes and apoptotic bodies. They have a function in intercellular communication but are also reported as a reservoir of biomarkers [8], contain lipids, RNA proteins, mRNAs and miRNAs [9]. Yan et al. conducted a study with patients suffering from mTLE-HS. The results showed the involvement of exosomal miRNAs as the seizure regulators and potent diagnostic biomarkers and therapeutic targets [10].

The aim of this review was the analysis of miRNA expression in children with epilepsy. The findings of animal models and adult patients can facilitate the design of studies in pediatric patients with the highest possible safety, comfort, and high reliability of the results obtained.

## 2. Materials and Methods

### 2.1. Search Strategy

A systematic search was conducted in the Pubmed database to identify the literature related to the involvement of miRNA in epilepsy. The following terms were used in the searching process: “miRNA”, “miRNAs”, “microRNA”, “microRNAs” and “epilepsy”. The search covered the period of 10 years back from 1 September 2020.

### 2.2. Selection Criteria

Manuscripts were reviewed for titles, abstracts, and the entire texts based on the following criteria. The inclusion criteria were as follows: (1) original papers; (2) epilepsy as a key topic of the paper; (3) studies comparing miRNA expression using PCR at different points in time or between groups without epilepsy, with epilepsy and refractory epilepsy; (4) manuscripts related to the influence of classical antiepileptic drugs on miRNA levels. The exclusion criteria were as follows: (1) reviews, case reports, methodological studies, editorials, commentaries, letters, hypotheses; (2) no available abstract; (3) manuscripts in a language other than English; (4) studies on cell lines; (5) material taken only from patients diagnosed with tuberous sclerosis complex (TSC), glioma, traumatic brain injury (TBI), cerebral ischaemia; (6) studies on changes in miRNA expression only on transgenic animals or those treated with novel methods aimed at modifying miRNAs.

### 2.3. Development of the Review

The analysis was conducted in the following steps. The first step was related to the analysis of selected papers based on titles and abstracts, the second step was connected with the analysis of full-text papers, and the last step included the analysis of the collected data. The data that met the inclusion criteria were entered into an Excel spreadsheet that included the following data: the title of a manuscript, animal/human model, group size, age, drugs, analyzed material, sampling time, PCR, type of miRNA and an increased or decreased level of miRNA expression in the group with epilepsy with the *p*-value.

## 3. Results

**Figure 1:**
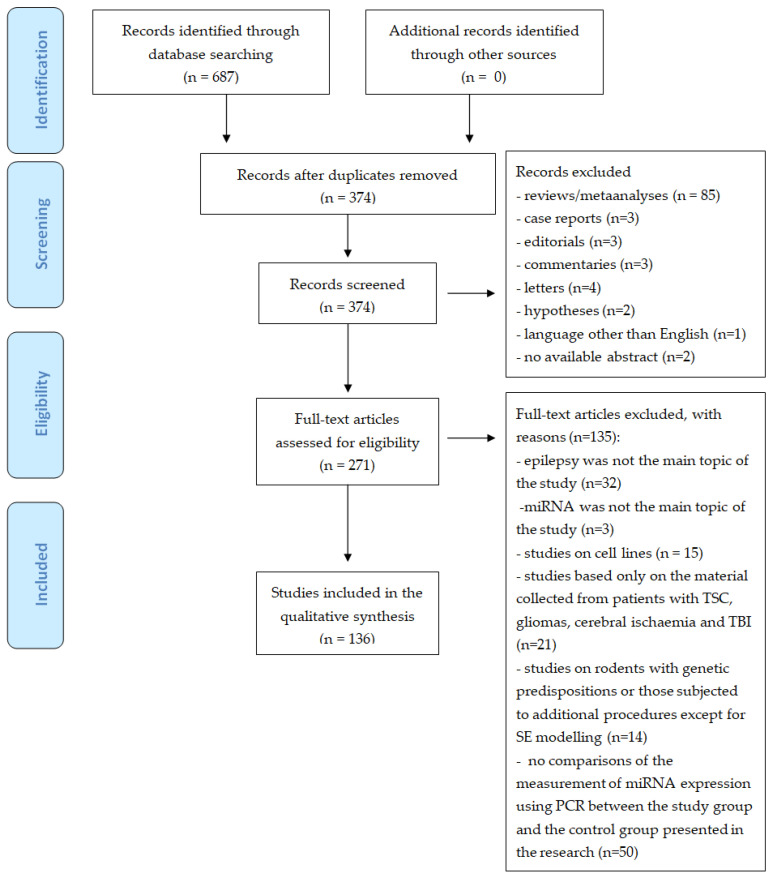
Flow diagram of PRISMA for the strategy research and selection processes for this review. TSC: tuberous sclerosis complex, TBI: traumatic brain injury, SE: status epilepticus.

The preliminary search of the database showed 687 studies, of which 271 were verified based on the entire manuscript. A total of 136 studies were finally included in the analysis (Figure 1). Identification of miRNA with altered expression based on polymerase chain reaction (PCR) was performed for each study that was included in the review. We identified upregulation or downregulation. We also identified whether the study group was comprised of humans or animals, and the type of biological material in which miRNA expression was measured. As a result, we selected the most frequently analyzed miRNAs. We paid attention to studies which simultaneously compared the expression of the same miRNAs in humans and animals or in different tissues or biofluids. All the data were intended to facilitate the design of miRNA expression studies in pediatric patients.

### 3.1. The Level of Expression of MiRNA in Children with Epilepsy

Based on our analysis, we identified only 10 manuscripts that included studies in children (Table 1). A total of 225 children with epilepsy were analyzed and 21 different miRNAs were studied. In more recent papers, there was growing tendency to use blood samples, while older studies were based on brain tissues collected intraoperatively from patients with refractory epilepsy. Temporal epilepsy was the most common diagnosis in the group of patients. Li et al. showed a significantly reduced expression of miR-15a-5p in children with epilepsy compared to healthy controls, which may indicate a potential significance in the pathogenesis of temporal lobe epilepsy (TLE). No significant differences were found between children with epilepsy and mesial sclerosis compared to children without these abnormalities. Additionally, no significant differences were observed between children with bilateral TLE compared to unilateral TLE. The change in expression did not correlate with the results of the EEG recordings or magnetic resonance imaging (MRI) studies [11].

Two studies included patients with cortical dysplasia. Tissues were collected for further examination during neurosurgery. The analysis of the miRNA expression profile was performed using microarrays. Next, miRNAs which showed an altered expression in the group with epilepsy was further verified using PCR. The results of both methods were consistent [12,13].

Wang et al. measured the serum level of mir-139-5p in three groups of patients, i.e., children with newly diagnosed epilepsy who were not given any medication, children with refractory epilepsy, and healthy children. The expression of mir-139-5p decreased in the group of patients with newly diagnosed epilepsy compared to the controls. The results of reduced expression in the group of drug-resistant patients were also statistically significant compared to newly diagnosed patients [14].

Three independent studies analyzed the expression of miR-146a. The results of these studies were consistent and miR-146a showed increased expression in the group with epilepsy [15,16,17].

### 3.2. Animal versus Human Models

Sampling at several time points in patients with epilepsy is still not very common. In our review, we identified four studies in which miRNA expression measurements were carried out at least twice. Brennan et al. analyzed plasma samples in patients with TLE which were taken during the seizure-free period (on admission and 24 h after the seizure). Based on their analysis, they found that the levels of most miRNAs were different between the two samples, which indicated their relationship to epilepsy rather than seizure. However, the levels of miR-142-5p were higher, which suggested that this miRNA may be transiently affected by acute seizure activity [21]. The same pattern of sampling was used by Raoof et al. who identified three miRNAs of high diagnostic significance [22]. In turn, Sun et al. collected serum during and after the seizure. They found overexpression of miR-378, miR-30a, miR-106b and miR-15a using RT-qPCR in the group of 90 patients [23]. Surges et al. collected blood at five different time points from patients with mesial temporal lobe epilepsy and compared changes in miRNA expression before and within 30 min, 3–6 h, 20–28 h and 3–6 days after bilateral convulsive seizures (BCS). Additionally, 215 miRNAs were significantly altered within 30 min after BCS compared to baseline levels. Further analysis showed that the change was transient and associated with BCS due to the fact that no significant differences were found at any later time point [24].

Studies on animal models showed the importance of more frequent sampling for a better understanding of epileptogenesis. Table 2 shows the studies in which simultaneous sampling was carried out in the animal and human groups. Additionally, sampling was done more than once in the animal model and the level of expression was measured using PCR. In Table 2, presented below, we distinguish only those publications that examined miRNAs verified in a pediatric group suffering from epilepsy.

Brennan et al. used three different animal models of status epilepticus. The serum samples were collected before the seizure, during epileptogenesis, and in chronic TLE. Five miRNAs potentially significant for epilepsy were identified. These miRNAs were verified in patients with TLE. Statistically significant results were found in three cases, which showed the same expression profile in each model. However, those authors we were unable to detect a single miRNA that would be characteristic at a given time point, but they noted that miRNA dysregulation was usually present in chronically epileptic mice [21].

The expression level of miR-155 was the subject of four studies. Korotkov et al. analyzed miR-155 expression in a rat model of SE at three different time points and found an increase in expression at all time points and all types of tissues [30]. The same miRNA was studied by Li et al. at four time points. The increase in miR-155 was noted in the first sample taken after the seizure, although the results were not statistically significant. The highest level of expression was found on day 7 and then it gradually decreased [31]. Huang et al. reported an increase in miR-155 level in CA3 after the seizure that peaked on day 14. The results were not statistically significant for CA1 [32].

Reschke et al. conducted an analysis of miR-134 expression levels in two different models of epilepsy and found an increase in expression 30 min after the administration of pentylenetetrazol in hippocampal samples of mice. Their results were consistent with those observed in the hippocampus of patients surgically treated for TLE. However, the study results conducted at the early time points (24 h, 4 days after the performed pathway stimulation) and at the late time point (14 days after the performed pathway stimulation) on a rat model of epilepsy were not statistically significant [27].

Zheng et al. was the only one to use cerebrospinal fluid to measure miR-219 and found a decrease in the levels of miR-219 in patients with epilepsy. Additionally, miR-219 levels decreased in hippocampal tissues in an animal model of epilepsy. However, changes in expression measured in cortical tissues showed no significant differences compared to the control group [33].

An additional animal model was present in 5 of 10 studies (Table 1), but four samples were collected several times [16,17,19,20]. Based on the literature, Ren et al. identified five miRNAs with possible significance for epilepsy. They confirmed altered expression of miR-132, miR-146a, miR-181a, miR-34a, and miR-124 in children with TLE and in a rat model. The most significant changes in the level of expression were found for miR-181. Therefore, the scope of the study was extended by the measurement at four time points, which resulted in significantly higher results in each time point compared to the control group [16].

The studies of Peng et al., Ashhab et al. and Omran et al. were based on a rat model of epilepsy in which three phases of development of mesial temporal lobe epilepsy were identified (acute phase associated with seizures, latent seizure-free phase and chronic phase associated with seizures). Of note, the expression of miRNA in children reflected the chronic stage in a rat model, and the results of both study groups were consistent [17,19,20].

### 3.3. Expression of MiRNAs in Blood and Other Biological Materials

To assess miRNA expression, it is easier to obtain blood samples than brain tissue. Therefore, we analyzed seven studies in which the same miRNAs were assayed in different materials of human origin in the same diseases (Table 3).

Interesting results were obtained by Antônio et al., who examined the expression of four miRNAs in patients with MTLE in blood and hippocampal tissue samples and two comparison groups, i.e., healthy control—blood samples and human tissues collected at autopsy. In blood samples, all miRNAs showed increased expression. However, in hippocampal tissues only miR-145 levels showed statistical significance compared to the control group but with the opposite direction of change than in the bioliquid [34].

Sun et al. measured the level of miR-129-2-3p and miR-935 in the brain tissue and plasma in two groups of patients with refractory temporal epilepsy and selected controls and achieved the same direction of change [38]. The same study regimen was used by Che et al. who analyzed the level of miR-323a-5p in patients with focal cortical dysplasia [37] and Fu et al. who studied miR-155 level in patients with TLE [35].

Li et al. found a decreased expression of miR-153 and miR-494 in brain tissue samples and a decreased level of miR-153 in plasma. Following neurosurgery, no significance of miR-494 was found in the same surgical patients with MTLE compared to the control group. The study was extended to a larger group of patients and matched controls. The results obtained from plasma samples were consistent [39].

Wang et al. evaluated hsa-miR-4521 level in patients with FCD and the control group due to an emergency neurosurgical procedure based on temporal cortex tissue and serum. In both samples, upregulated expression of hsa-miR-4521 was found in patients with epilepsy [40].

### 3.4. Impact of Drugs on MiRNA Levels

The opinions on the results of the impact of antiepileptic therapy on a reliable miRNA expression level are not consistent. Brennan et al. analyzed the effect of carbamazepine and diazepam on miRNA expression at two time points, before and after 3 days of therapy. None of the five miRNAs showed a statistically significant change. A different result was achieved when antiepileptic drugs were used. These drugs (i.e., antisense oligonucleotide inhibitors, also known as antagomirs) directly targeted miRNAs and hence altered the level of some of the miRNAs [21]. The same set of drugs used for 3 days in a mouse model of epilepsy was used by Raoof et al. The expression levels of miR-27a-3p, miR-328-3p, and miR-654-3p showed no changes in plasma after therapy [22]. Different results were obtained by Wang et al. who studied the expression level of miR-134 in patients with newly diagnosed epilepsy. Significantly higher levels were found only in patients with severe epilepsy compared to the control group. After one month of treatment with valproic acid, the mean level of miR-134 was significantly decreased in patients with severe epilepsy (*p* < 0.05) [41].

Haenisch et al. administered phenobarbital to rats twice a day for 2 weeks, the levels of the miRNAs measured did not change significantly [42]. In addition, there is evidence of the potential use of miR-155 as a biomarker for the efficacy of antiepileptic therapy [35].

### 3.5. The Most Important MiRNA in the Context of Epilepsy

Based on our analysis of the studies, we identified potential miRNAs related to epilepsy. However, we paid attention to miRNAs of the most diagnostic and prognostic significance.

Table 4 shows miRNAs whose expression correlates with the clinical picture.

We also analyzed miRNAs as potential biomarkers for refractory epilepsy (Table 5), diagnostic biomarkers (Table 6) and prognostic biomarkers (Table 7). To identify the studies with the highest importance, we focused on studies that used the analysis of receiver operating characteristic (ROC) curves. The closer the area under the curve (AUC) to 1, the higher the sensitivity and specificity of a diagnostic marker.

Leontariti et al. showed elevated serum levels of miR-146a and miR-134 in drug-resistant patients and found that they were a risk factor for developing refractory epilepsy independently of other clinical aspects such as family history, gender, age at the first seizure, or duration of epilepsy. It is suggested that patient care combined with the assessment of the levels of these miRNAs could improve early prognosis in this group of patients [47]. The inhibitory effect on the development of drug resistance by targeting MRP1 was attributed to miR-139-5p [14].

Chen et al. identified miR-434-3p and miR-133a-3p as potential circulating biomarkers of epileptogenesis [53]. However, Raoof et al. considered miR-328-3p (AUC = 0.9) and miR-27a-3p (AUC = 0.88) potential biomarkers of seizure [22].

## 4. Discussion

Epilepsy is a common neurological condition in pediatric patients [54], and making an accurate diagnosis is a challenge in this age group. Uldall et al. conducted a study verifying the diagnosis of epilepsy in a group of 223 children, of whom 86% were on antiepileptic drugs. The review of the clinical picture (including EEG recordings) showed that 39% of children were found not to have epilepsy and 15% of children had their medication tapered off. The study indicated a large scale of misdiagnosis [55]. Young children are particularly susceptible to seizures, especially SE. The etiology of this phenomenon is multifactorial. Internal and external factors are distinguished. The former include, e.g., immaturity of intrinsic endogenous networks which lead to seizure termination, increase in local excitability, whereas the latter include hypoxic-ischemic insult, fever, or inflammation [56]. Epileptogenesis is a complex process that is initiated by traumatic brain injury, which results in many cellular and molecular changes that lead to spontaneous seizures [57]. In the treatment of epilepsy, it is crucial to reduce the seizure duration and its frequency, which is associated with neuroprotection [11]. However, about 3 in 10 patients are drug-resistant. According to the definition proposed by the International League Against Epilepsy (ILAE), the above is related to the failure of therapy with two well-tolerated, appropriately selected antiepileptic drugs. Understanding the mechanism of epileptogenesis at an early stage could help to develop a therapy that would prevent or modify these changes [20].

The above problems affect the intensive search for new diagnostic, prognostic and therapeutic methods. As described above, there is a wide database of miRNAs which undergo expression changes in patients with epilepsy. Using them as diagnostic biomarkers would not only allow for a more accurate diagnosis of epilepsy, but it would also accelerate the diagnosis time and reduce costs, thus improving the quality of life of patients and their families. It has been proven that miRNA is stable in various biological materials and it reflects the damage to the central nervous system (CNS) [58]. Determination of miRNA levels in blood is an easy, quick, accessible, and slightly invasive method that offers new perspectives. Firstly, it is possible to conduct more extensive studies on the pediatric population. Due to the dynamic expression pattern, sampling at more than one time point provides researchers with information which of the dysregulated miRNAs can be a potential marker of acute seizure (change in the miRNA level after seizure) or epilepsy (before and after a seizure, the miRNA level will not change significantly) compared to the control. Greater availability of the comparative material from healthy controls will objectify the results compared to the studies in which hippocampal tissues were used. These tissues were collected during surgical treatment of epilepsy. It will also be possible to compare them with the control group in which brain tissues were obtained from neurosurgical procedures performed due to other indications or from post-mortem examinations. Roncon et al. compared miRNA expression in three groups, i.e., brain tissues intraoperatively collected from patients with epilepsy, brain tissues of patients with epilepsy taken during post-mortem examinations, and brain tissues from post-mortem examinations from patients without a history of seizures. Hierarchical clustering showed that the samples obtained from post-mortem examinations of patients with epilepsy were segregated with the other post-mortem tissues. Despite a small study group, the origin of the tissue may be more significant than the underlying pathology [59]. In turn, a study on a mouse model of epilepsy (in which the autopsy delay was intentionally induced) showed no significant differences in miRNA levels measured immediately, 4 h and 8 h post-mortem [60]. Bencurova et al. presented similar results of miRNA stability in autopsy samples [61]. To our knowledge, the usefulness of miRNA determination in saliva samples in patients with epilepsy has not been investigated, but studies conducted in a pediatric group with a diagnosis of spectrum autism disorder presented promising results for the use of this bioliquid [5,62].

All the presented reports testify to the need to standardize the studies on the use of miRNA. Liguori et al. conducted a study on a pediatric group of patients suffering from multiple sclerosis (MS). The researchers pointed out the limitations of the use of microarrays and the possibilities offered by the use of HighThroughput Next-generation Sequencing (HT-NGS), which enables miRNA and mRNA profiling [63].

The part of miRNAs can be considered prognostic biomarkers of epilepsy, drug resistance, surgical prognosis and effectiveness of therapy. Predicting resistance to treatment is of crucial importance, since through a precisely selected, effective and timely therapy, patients can be protected against long-term complications of epileptic seizures. New epileptic subtypes with a characteristic course and prognosis may be identified using miRNAs. Animal studies are also promising, as they modify the expression of miRNAs (through innovative methods) with a simultaneous follow-up of seizures or histopathological changes. Therefore, miRNAs are potential molecular targets for drug design. An innovative approach modifying the level of miRNAs is the use of antagomirs (antisense oligonucleotides that bind and block miRNAs) or agomirs (miRNA imitators that increase its level). Of the entire pool of miRNAs that have been linked to epilepsy, only a small part have been manipulated. The use of an antagomir to suppress the expression of miR-134 resulted in prolonged seizure suppression and neuroprotective effects [27,64,65], while the use of a nasally administered antagomir to suppress miR 155-5p reduced the severity of seizures, prolonged the delay to generalized seizures, and increased the percentage of seizure-free animals by 20% [66]. The use of miR-34a antagomir in the Hu et al. study contributed to increased neuronal survival and had a neuroprotective effect [67], while the Sano et al. study showed little effect on apoptotic changes and did not reduce neuronal death in mouse models of moderate and severe epileptic condition [68]. Other researchers have used the miR-181, miR-132, miR-155-5p antagomirs to achieve a neuroprotective effect and reduce seizure-induced neuronal death [16,32,69]. Less research has been devoted to the use of agomirs, Wang et al. evaluated the effect of injecting miR-146a agomir and miR-146a antagomir in a group of rats in which SE was induced with lithium chloride and pilocarpine. The rats that received the antagomir presented longer seizures and shorter latency periods; in the group treated with agomir, the results were reversed [70]. Zhang et al. indicated that suppressing the expression of miR-146a may improve drug resistance and weaken pathological changes in patients with refractory epilepsy [71]. In the Iori et al. study, the use of miR-146a agomir reduced seizure severity and neuronal excitability [72].

The research on miRNAs is of growing interest. The fact that miRNA names depend on the order in which they are detected and are assigned according to the numerical system proves how large our database is [73]. The exception to this rule is the let-7 family. The data presented in the above review are only a fragment of the studied miRNAs, which have been associated with epilepsy and provide a basis for further research on the function of these molecules in the mechanisms of epileptogenesis. However, the discussion of all miRNAs exceeds the possibilities of the presented review, but we would like to present some of the possible options for regulating gene expression in epilepsy.

The miR-146a has been associated with the IL-1/Toll-like receptor pathway (IL-1R1/TLR4), its activation results in a lower seizure threshold and neuronal hyperactivity, which promotes epileptogenesis. The experimental use of miR-146a agomir lowers the levels of two key proteins: IRAK-2 and TRAF-6, which mediate IL-1R1/TLR4 signaling [72].

Alsharafi et al., using the experimental TLE model, showed that the miR-139-5p targets the enthralling alpha 2 subunit of the neuronal NMDA receptor NMDA-NM2A. The role of NMDA receptors is to participate in the neurotransmission and plasticity of neurons, and their activation is associated with the generation and maintenance of seizures and death of neurons in the hippocampus area [29]. Another target gene for miR-139-5p is multidrug resistance-associated protein 1 (MRP1). Modifications involving down-regulation of MRP1 or overexpression of miR-139-5p may have neuroprotective effects by increasing the survival of neurons, alleviating their damage and reducing apoptosis [14].

MiR-155 has been found to be a strong regulator of Sestrin-3, it is a gene associated with oxidative stress prevention, and the resulting interdependence may provide a molecular basis for the development of seizures during brain injury [32].

MiR-34a and miR-181a affect apoptosis-related genes such as bcl-2 and caspase-3 [16,67,74]. In turn, the combined action of miR-137 and miR-124 regulates the expression of BCL2L13 (atypical BCL2 protein), which controls the release of cytochrome C and caspase-3 activity in nerve stem/progenitor cells and may affect early neurogenic response after the onset of a seizure [75].

The EpimiRBase is a database that provides up-to-date information on changes and the role of miRNAs in epilepsy. In addition, we have access to other databases such as miRTarBase, TargetScan to search for potential miRNA targets.

Despite the enormous prospects and promising study results, the route of miRNAs from the laboratory to widespread clinical use is still very long.

## 5. Limitations

This review has several limitations, including the small number of studies on the pediatric population which considers a known genetic background of epilepsy, the comparison of the results of miRNA expression in the group with epilepsy versus healthy controls, autopsy samples or samples collected during major neurosurgical procedures, an uncertain effect of antiepileptic drugs on changes in miRNA expression and the heterogeneity of the study groups.

## 6. Conclusions

MiRNAs are a promising novel diagnostic and predictive tool and a potential therapeutic target. Further studies on the pediatric population are warranted, because still little is known about this age group. In addition, despite a significant database of miRNAs with dysregulated expression, their relationship to epileptogenesis remains unclear.

## Figures and Tables

**Table 1:** Expression of selected miRNAs measured in a group of pediatric patients diagnosed with epilepsy.

	Children Diagnosed with Epilepsy			Control Children				MiRNA Expression in Children with Epilepsy	
References	Patients	Number of Patients	Age (Years)	Controls	Number of Controls	Age (Years)	Samples	Up	Down
Li, N. et al. 2020 [11]	TLE	63	9.81 ± 2.79	healthy	67	10.13 ± 2.46	serum		miR-15a-5p
Elnady, H.G. et al. 2019 [15]	epilepsy	30	5–15	Healthy	20	5–15	plasma	miR-146a	
								miR-106b	
Wang, L. et al. 2020 [14]	refractory epilepsy	26		NDE	35		serum		miR-139-5p
	NDE	35		traumatic brain injury or cerebrovascular malformation	20				miR-139-5p
Wu, X. et al. 2019 [18]	TLE	15	11.2 ± 2.6	normal	15	10.6 ± 3.7	hippocampal Area CA3	miR-135a-5p	
Ren, L. et al. 2016 [16]	TLE	25			11		brain tissues	miR-181a	
								miR-132	
								miR-146a	
								miR-34a	
								miR-124	
Li, L. et al. 2016 [12]	FCD type II B	5	50–112 months				brain tissues	let-7f-1-3p	miR-6511b-5p
								miR-1281	miR-6862-5p
								miR-940	
								miR-1825	
Lee, J.Y. et al. 2014 [13]	cortical dysplasia	8	1–15	deep-seated lesions	3	2–13	brain tissues	miR-21	
								miR-155	
								miR-130b	
								miR-193b	
								miR-199b	
Ashhab, M.U. et al. 2013 [19]	MTLE	8	8–13	no history of any brain disease	8	6–13	hippocampal tissues	miR-155	
Peng, J. et al. 2013 [20]	MTLE	5	8–12	no history of any brain disease	5	8–12	hippocampal tissues	miR-124	
								miR-134	
								miR-132	
								miR-21	
Omran, A. et al. 2012 [17]	MTLE	5	8–12	no history of any brain disease	5	8–12	hippocampal tissues	miR-146a	

NDE: Newly Diagnosed Epilepsy, FCD: Focal Cortical Dysplasia, MTLE: Mesial Temporal Lobe Epilepsy.

**Table 2:** Expression of selected miRNAs measured in animal models of epilepsy and in group of patients diagnosed with epilepsy.

References	Experimental Animals and Epilepsy Induction	Tissue	Time Points	miRNA Studied	Level of Expression in a Group with Epilepsy	Patients	Tissue	Level of Expression in a Group with Epilepsy
Peng, J. et al. 2013 [20]	PILO-induced SE in a rat model	HP	2 h post SE (AP)	miR-124	up	MTLE	HP	up
				miR-134	up			up
				miR-132	up			up
				miR-21	up			up
			3 weeks post SE (LP)	miR-124	ns			
				miR-134	ns			
				miR-132	up			
				miR-21	down			
			8 weeks post SE (CP)	miR-124	up			
				miR-134	up			
				miR-132	up			
				miR-21	up			
Korotkov, A. et al. 2020 [25]	tetanic stimulation-induced SE in a rat model (50 Hz)	DG	1 day post SE (AP)	miR-132	up	TLE-HS	HP	up
			1 week post SE (LP)		ns			
			3–4 months post SE (CP)		ns			
		CA1	1 day post SE (AP)		ns			
			1 week post SE (LP)		ns			
			3–4 months post SE (CP)		ns			
Guo, J. et al. 2014 [26]	lithium-PILO-induced epilepsy in a rat model	HP	24 h post SE	miR-132	up	TLE	temporal neocortex	down
			72 h post SE		ns			
			7 d post SE		up			
			14 d post SE		ns			
			30 d post SE		ns			
			60 d post SE		ns			
Reschke, C.R. et al. 2017 [27]	PTZ model of generalized tonic-clonic seizures in mice	cortex	30 min after PTZ injection	miR-134	ns	TLE	HP	up
		HP			up			
	PPS model of epilepsy in rats	HP	24 h and 4 days after PPS		ns			
			14 days after PPS		ns			
Alsharafi, W. et al. 2015 [28]	PILO-induced SE in a rat model	HP	2 h post SE (AP)	miR-135a	up	TLE	HP	up
			2 months post SE (CP)		up			
Alsharafi, W.A. et al. 2016 [29]	PILO-induced SE in a rat model	HP	1 day after SE (AP)	miR-139-5p	down	TLE	HP	down
			7 days after SE (LP)		ns			
			60 days after SE (CP)		down			
Omran, A. et al. 2012 [17]	lithium-PILO-induced SE in a rat model	HP	2h post SE (AP)	miR-146a	ns	MTLE	HP	up
			3 weeks post SE (LP)		up			
			8 weeks post SE (CP)		up			
Korotkov, A. et al. 2018 [30]	tetanic stimulation-induced SE in a rat model (50 Hz)	brain tissue: DG, CA1, PHC	1 day post SE (AP)	miR-155	up	TLE-HS	HP	up
			1 week post SE (LP)		up			
			3–4 months post SE (CP)		up			
Li, T.R. et al. 2018 [31]	KA-induced SE in a rat model	HP	2 h after post SE (AP)	miR-155	ns	TLE-HS	HP	up
			7 days post SE (LP)		up			
			21 days post SE (LP)		up			
			60 days post SE (CP)		up			
Huang, L.G. et al. 2018 [32]	PILO-induced TLE in a rat model	CA1	0 day post-SE	miR-155	ns	TLE	CA1, CA3	up
			1 day post-SE		ns			
			14 days post-SE		ns			
			30 days post-SE		ns			
			60 days post-SE		ns			
		CA3	0 day post-SE		ns			
			1 day post-SE		up			
			14 days post-SE		up			
			30 days post-SE		up			
			60 days post-SE		up			
Ashhab, M.U. et al. 2013 [19]	lithium-PILO-induced SE in a rat model	HP	2 h post SE (AP)	miR-155	up	MTLE	HP	up
			3 weeks post SE (LP)		ns			
			8 weeks post SE (CP)		up			
Ren, L. et al. 2016 [16]	lithium-PILO-induced SE in a rat model	HP	24 h post SE	miR-181a	up	TLE	brain tissues	up
			7 days post SE		up			
			14 days post SE		up			
			3 months post SE (TLE)		up			
				miR-132	up			up
				miR-146a	up			up
				miR-34a	up			up
				miR-124	up			up

KA: kainic acid, PILO: pilocarpine, PPS: perforant pathway stimulation, PTZ: pentylenetetrazole, DG: dentate gyrus, CA1: cornu Ammonis 1, PHC: parahippocampal cortex, HP: hippocampus, CA3: cornu Ammonis 3, AP: acute phase, LP: latent phase, CP: chronic phase, TLE-HS: Temporal Lobe Epilepsy with Hippocampal Sclerosis, ns: no significance.

**Table 3:** MicroRNA expression measured in different biological materials in patients with the same clinical diagnosis.

References	Patients	Number	Controls	Number	Samples	Method	miRNA Expression in Epileptic Patients			
							Up	Down	*p*-Value	Not Significant
Antônio, L.G.L. et al. 2019 [34]	MTLE-HS	20	no neurological or psychiatric medical history	9	hippocampal	RQ-PCR		miR-145	*p* = 0.02	miR-199a
										miR-1183
										miR-181c
			healthy control	10	blood		miR-145		*p* = 0.005	
							miR-181c		*p* = 0.03	
							miR-199a		*p* = 0.01	
							miR-1183		*p* = 0.001	
Fu, H. et al. 2019 [35]	TLE	12	no history of epilepsy	11	brain samples	qRT-PCR	miR-155		*p* < 0.05	
	epilepsy	40	no history of epilepsy	40	plasma		miR-155		*p* < 0.001	
Gong, G.H. et al. 2018 [36]	MTLE	22	no history of epilepsy or seizures	20	temporal cortex	qRT-PCR		miR-153	*p* < 0.01	
					plasma			miR-153	*p* < 0.01	
Che, N. et al. 2017 [37]	FCD	9	hypertensive cerebral hemorrhage and no reported neurological illness	8	cortical samples	qRT-PCR	miR-323a-5p		*p* = 0.012	
		30	healthy control	23	plasma		miR-323a-5p		*p* = 0.0320	
Sun, Y. et al. 2016 [38]	TLE	13	no history of neurological diseases	13	cortical samples	qRT-PCR	miR-129-2-3p		*p* < 0.0001	miR-935
		25		25	plasma		miR-129-2-3p		*p* = 0.0008	miR-935
Li, Y. et al. 2016 [39]	MTLE	32	no history of epilepsy or seizures	18	temporal cortex	RT-qPCR		miR-153	*p* < 0.001	miR-494
					plasma			miR-153	*p* < 0.001	miR-494
		56	healthy control	101	plasma			miR-153	*p* < 0.001	miR-494
Wang, X. et al. 2016 [40]	TLE, FCD	9	acute intracerebral hematoma, no neurological illness associated with epilepsy	8	temporal cortex	RT-qPCR	hsa-miR-4521		*p* = 0.001	
					serum		hsa-miR-4521		*p* = 0.0145	

**Table 4:** Expression of miRNAs whose correlates with the clinical characteristics.

References	miRNA	Clinical Characteristics
Elnady, H.G. et al. 2019 [15]	miR-146a	age (*p* = 0.007)
Organista-Juárez, D. et al. 2019 [43]	miR-1260	age (*p* = 0.018)
	miR-1298	age (*p* = 0.022)
	miR-146a	seizure frequency (*p* = 0.009)
		number of antiepileptic drugs (*p* = 0.03)
	miR-451	number of antiepileptic drugs (*p* = 0.046)
Shen, C.H. et al. 2019 [44]	miR-145-5p	earlier age at epilepsy onset (*p* = 0.024) seizure frequency (*p* = 0.020) past history (head trauma, encephalitis) (*p* = 0.014)
Gong, G.H. et al. 2018 [36]	miR-153	seizure frequency (*p* = 0. 018) Engel classification (*p* < 0. 01)
Huang, L.G. et al. 2018 [32]	miR-155-5p	hippocampal sclerosis (*p* = 4.03 × 10^−5^) Engel classification (*p* = 3.54 × 10^−5^) seizure frequency (*p* = 0.028)
Wang, X. et al. 2017 [41]	miR-134	seizure severity (*p* = 0.016 moderate seizure group, *p* = 0.003 severe seizure group)
Yan, S. et al. 2017 [10]	miR-8071	disease duration (*p* = 0.0073) seizure frequency (*p* = 0.0316)
Che, N. et al. 2017 [37]	miR-323a-5p	disease duration (*p* = 0.014) seizure frequency (*p* = 0.043) poor prognosis (*p* = 0.028) effectiveness of surgery (*p* = 0.005)
Surges, R. et al. 2016 [24]	miR-143-3p, miR-145-5p	total seizure duration
Sun, J. et al. 2016 [23]	miR-30a	seizure frequency (*p* < 0.01)
An, N. et al. 2016 [45]	miR-106b	seizure severity using the National Hospital Seizure Severity Scale (NHS3)
Sun, Y. et al. 2016 [38]	miR-129-2-3p	Engel classification (*p* = 0.005) seizure frequency (*p* = 0.027)
Wang, J. et al. 2015 [46]	miR-301a-3p	seizure severity using the National Hospital Seizure Severity Scale (NHS3) (*p* = 6.2 × 10^−9^)

**Table 5:** miRNA as a potential biomarker of drug resistance.

References	miRNA	AUC
Leontariti, M. et al. 2020 [47]	miR-146a	0.640
	miR-134	0.617
Shen, C.H. et al. 2019 [44]	miR-145-5p	0.632
Wang, X. et al. 2016 [40]	miR-4521	0.718
Li, Y. et al. 2016 [39]	miR-153	
Sun, Y. et al. 2016 [38]	miR-129-2-3p	0.778
Wang, J. et al. 2015 [46]	miR-301a-3p	0.893

**Table 6:** miRNA as a potential diagnostic biomarker for epilepsy.

References	miRNA	Diagnostic Marker	Sensitivity	Specificity	AUC
Ioriatti, E.S. et al. 2020 [48]	miR-328-3p	MTLE-HS	89.30%	90.90%	0.935
Brennan, G.P. et al. 2020 [21]	miR-93a-5p, miR-199a, miR-574-3p	TLE			0.88–0.86
Martins-Ferreira, R. et al. 2020 [49]	miR-146a, miR-155, miR-132	GGE	73%	80%	0.850
Li, N. et al. 2020 [11]	miR-15a-5p	TLE	82.50%	88.10%	0.908
Shen, C.H. et al. 2019 [44]	miR-145-5p	MTLE			0.829
Elnady, H.G. et al. 2019 [15]	miR-106b	epilepsy	80%	80%	0.885
	miR-146a		73.70%	60%	0.763
Raoof, R. et al. 2018 [22]	miR-27a-3p	TLE			0.630
		GGE			0.730
	miR-328-3p	TLE			0.630
	miR-654-3p	TLE			0.870
		GGE			0.720
	miR-27a-3p, miR-328-3p, miR-654-3p	TLE			0.640
		GGE			0.740
Raoof, R. et al. 2017 [50]	miR-451a, mir-21-5p	TLE and SE			0.850
	miR-19b-3p, miR-21-5p, miR-451a				0.830
Avansini, S.H. et al. 2017 [51]	miR-134	MTLE	75%	58%	0.671
Yan, S. et al. 2017 [10]	miR-8071	MTLE-HS	83.33%	96.67%	0.9316
An, N. et al. 2016 [45]	miR-106b	epilepsy			0.887
	miR-146a				
Wang, J. et al. 2015 [52]	miR-106b-5p	epilepsy	80.30%	81.20%	0.882

GGE: genetic generalized epilepsies.

**Table 7:** miRNA as a potential prognostic biomarker of epilepsy.

References	miRNA	Prognostic Biomarker	AUC
Ioriatti, E.S. et al. 2020 [48]	miR-654-3p	MTLE-HS	0.736
Shen, C.H. et al. 2019 [44]	miR-145-5p	MTLE	0.829

## Data Availability

Publicly available datasets were analyzed in this study. This data can be found here: https://pubmed.ncbi.nlm.nih.gov/.

## Abbreviations

AP	acute phase
CA1	cornu Ammonis 1
CA3	cornu Ammonis 3
CP	chronic phase
CSF	cerebrospinal fluid
DG	dentate gyrus
FCD	Focal Cortical Dysplasia
GGE	Genetic Generalized Epilepsies
HP	hippocampus
KA	kainic acid
LP	latent phase
MTLE	Mesial Temporal Lobe Epilepsy
MTLE-HS	Mesial Temporal Lobe Epilepsy with Hippocampal Sclerosis
NDE	Newly Diagnosed Epilepsy
NS	no significance
PHC	parahippocampal cortex
PILO	pilocarpine
PPS	perforant pathway stimulation
PTZ	pentylenetetrazole
SE	status epilepticus
TLE	Temporal Lobe Epilepsy
TLE-HS	Temporal Lobe Epilepsy with Hippocampal Sclerosis
